# Machine Learning Approach to Raman Spectrum Analysis of MIA PaCa-2 Pancreatic Cancer Tumor Repopulating Cells for Classification and Feature Analysis

**DOI:** 10.3390/life10090181

**Published:** 2020-09-05

**Authors:** Christopher T. Mandrell, Torrey E. Holland, James F. Wheeler, Sakineh M. A. Esmaeili, Kshitij Amar, Farhan Chowdhury, Poopalasingam Sivakumar

**Affiliations:** 1 Department of Physics, Southern Illinois University Carbondale, Neckers 483-A, 1245 Lincoln Drive, Carbondale, IL 62901, USA; chrismandrell@siu.edu (C.T.M.); torrey.holland@siu.edu (T.E.H.); james.wheeler@siu.edu (J.F.W.); 2 Department of Mechanical Engineering and Energy Processes, Southern Illinois University Carbondale, 1263 Lincoln Drive, Carbondale, IL 62901, USA; sesmaei@siu.edu (S.M.A.E.); k.amar@siu.edu (K.A.); farhan.chowdhury@siu.edu (F.C.)

**Keywords:** tumor repopulating cell (TRC), support vector machine (SVM), k-nearest neighbor (kNN), principal component analysis (PCA), Raman spectroscopy, pancreatic cancer

## Abstract

A machine learning approach is applied to Raman spectra of cells from the MIA PaCa-2 human pancreatic cancer cell line to distinguish between tumor repopulating cells (TRCs) and parental control cells, and to aid in the identification of molecular signatures. Fifty-one Raman spectra from the two types of cells are analyzed to determine the best combination of data type, dimension size, and classification technique to differentiate the cell types. An accuracy of 0.98 is obtained from support vector machine (SVM) and k-nearest neighbor (kNN) classifiers with various dimension reduction and feature selection tools. We also identify some possible biomolecules that cause the spectral peaks that led to the best results.

## 1. Introduction

Pancreatic adenocarcinoma, accounting for about 93% of pancreatic cancers, is one of the most aggressive and deadly cancers, with a 5 year survival rate of about 9%, which is primarily due to drug resistance or metastasis, as well as late-stage diagnoses [1,2,3]. Drug resistance or metastasis is attributed to the presence of tumor repopulating cells (TRCs) or stem cell-like cancer cells [4,5,6], which are a subpopulation of self-renewing cells within malignant tumors that are highly tumorigenic [7,8]. TRCs are still not fully understood, and no known reliable biomarkers are available to detect or track these self-renewing cells. Gaining knowledge of TRCs can assist in a better understanding of tumorigenesis and tumor resistance to therapy [9].

Nondestructive, minimally invasive tools are needed to distinguish between the highly tumorigenic TRCs, normal cancer cells, and healthy tissues with a high sensitivity, specificity, and reliability. For this reason, clinical spectroscopy techniques have been growing in popularity amongst researchers, especially Raman spectroscopy [10]. This is because Raman spectroscopy is a nondestructive technique requiring little or no sample preparation that can be applied in vivo or in vitro. Moreover, it provides qualitative and quantitative information about the chemical structure and composition of the sample via an analysis of the inelastic scattering from discrete vibrational and rotational transitions in molecules [10,11].

Machine learning originated from artificial intelligence research and is a rapidly growing field that shows great promise in medical research, due to its ability to detect patterns in complex datasets that humans would likely miss [12]. Researchers tested the feasibility of using machine learning algorithms to predict the probability of pancreatic cancer patients relapsing by following resection with prior induction polychemotherapy and chemoradiation. The algorithms used were logistic regression, decision tree, random forest, support vector machine (SVM), and k-nearest neighbors (kNN), and they attained a highest accuracy of 71% using a logistic regression [13].

Here, the supervised machine learning classifiers, kNN and SVM, will be utilized. kNN locates a user-selected number (k) of the nearest neighbors to a sample, and classifies the sample as belonging to the same class as its neighbors by taking a majority vote of those k neighbors [12]. Note that kNN does not build a model that can be referred to later but must compare each new instance to the full training set [12]. This can lead to heavy computational demands, but kNN has been shown to do relatively well in a high dimension space with a small sample size, making it of particular interest in these studies [14,15,16].

SVMs utilize linear algebra manipulations to locate a hyperplane that maximally separates the classes in a sample space. A sample’s support vector represents its relationship to the hyperplane, and the SVM simply identifies a new sample’s support vector and classifies it based on that information. SVMs build a model that can be quickly referred back to, so it can be computationally expensive in the model building process, but the later classification is relatively computationally inexpensive. A powerful tool utilized by SVMs is the kernel trick. A kernel is a mathematical manipulation of the sample space than can distort the space in such a way as to make it easier, or in some cases merely possible, to find a hyperplane to separate the classes. Examples of standard kernels are linear-, polynomial-, sigmoid-, and radial-based (e.g., Gaussian) [12]. All these kernels have been applied to the data in this study for comparison and optimization.

In this work, we applied machine learning and dimension reduction techniques to the Raman spectra of TRCs and parental control cells from the MIA PaCa-2 pancreatic cancer cell line to search for reliable diagnostic molecular signatures and to determine the molecules that lead to the best differentiation of these types of cells. Combining machine learning with Raman spectroscopy to delineate between TRCs and control pancreatic cancer cells is quite novel if not a first in this field.

## 2. Methodology

### 2.1. Feature Selection and Classification

One of the most significant problems encountered during the classification of complex spectroscopic data, including Raman spectra, in biological samples is the overfitting or underfitting of the models due to the “large dimension-small sample size” problem [15,16,17,18]. While a medical study might produce a couple of hundred samples, each wavenumber that is measured during Raman data collection serves as a dimension in the sample space that could consist of thousands of dimensions. Dimension reduction is a technique to address this problem that is actually two different techniques: dimension elimination and dimension selection [16,19]. It plays a vital role in the performance of the classification algorithms.

Dimension elimination is the process of discarding unwanted data with minimal analysis. This could involve eliminating quiet or always noisy regions of a spectrum. For example, in biological samples, the Raman fingerprint region of the skeletal vibrations tends to lie between the wavenumbers 600 and 1800 cm^−1^, with the high energy signals of biologicals appearing in the region of 2500–3400 cm^−1^ due to the independent motions of hydrogen atoms [20]. The O-H bond of water tends to appear as a broad peak around 3350 cm^−1^ [21], and the region between 1800 and 2500 cm^−1^ tends to be relatively silent [20], so selective targeting of the Raman range is possible. Therefore, instead of scanning from 250 to 3500 cm^−1^ for ~2950 dimensions, selective scanning from 600 to 1800 cm^−1^ and 2500–3400 cm^−1^ provides the pertinent information while limiting the dimension size to ~1900 (dimension count based on the approximation of wave numbers acquired from a diffraction grating with a groove density of 600 g/mm). It is also possible to reduce the dimensions by only considering the values of the spectrum that coincide with the local maxima (i.e., peaks). Both techniques are utilized in this work, and the peaks vs. continuous spectrum results are compared.

Dimension selection is a more analytical process and requires a logical analysis of the relevance of each feature to determine which dimensions have the highest correlation to the class of the sample. There are many techniques already in use or in development to accomplish this task. However, for this work, only four—T-statistic (*t*) [14], MIT correlation (*w*) [14], RELIEF (*r*) [18], and principal component analysis (PCA) [22,23], as seen in Table 1—were considered.

The *t* and *w* feature selection methods provide statistical measures of each wavenumber’s (dimension’s) correlation to the class in which the sample belongs. Each feature (dimension) is given a score, and the features with the highest scores are chosen [14].

The *r* technique from Marchiori, 2005, uses a machine learning algorithm to determine the nearest neighbor to each sample from both classes for a specific feature. Each dimension is given a weight based on its distance to its nearest neighbor from each class with respect to the feature in question. The higher the *r*-score, the better the feature is at maximizing interclass distance and minimizing the intraclass distance [18].

PCA reshapes a sample space with dependent variables into an independent space that still contains all the total variances of the original space. It accomplishes this by finding the eigenspace of the covariance matrix of the original samples. It is useful in that it creates a linearly independent basis, and in that, most of the variance usually occurs over many fewer variables. By choosing the number of principal components or the percentage of the overall variance desired, PCA can be manipulated to provide a useful representation of the original data in far fewer dimensions [22,23]. Each of these techniques was run individually for a varied set of features, and PCA was also run in combination with *t*, *w*, and *r* (e.g., PCA on 4, 5, 10, 15, 150 dimensions chosen by *t, w*, and *r*).

### 2.2. Accuracy Metrics

The accuracy reported in this study was determined using the results of a k-fold cross-validation (CV) scheme, where the dataset was broken up into k subsets and the classifier ran k iterations. At each iteration, a different subset was held out for testing while the rest of the subsets were used to train the model, and the results from each iteration were averaged to provide the CV accuracy score and standard deviation [15]. The Python package, sklearn.metrics, was used to acquire these CV scores.

Model overfitting is a common problem in the classification of complex datasets [24]. This is when the model “memorizes” the training data and is therefore not generalizing the data. One way to evaluate if a model is overfitting is to compare the training accuracy to the testing accuracy. Overfitting occurs when the training accuracy is higher than the testing accuracy [25]. For these reasons, the CV training accuracy was also used in this analysis.

## 3. Experimental Approach

### 3.1. Sample Preparation

MIA PaCa-2 cells, purchased from ATCC (ATCC^®^ CRL-1420), were routinely cultured on gelatin (Sigma-Aldrich) coated 6-well tissue-culture dishes (Eppendorf) with a complete medium containing 10% fetal bovine serum and 2.5% horse serum at 37 °C with 5% CO_2_. Cells were passaged when they reached 80–90% confluency. For experiments, cells were trypsinized, centrifuged for 3 min, and were resuspended in a 1 mL complete culture medium. Fibrin gel of 90 Pa stiffness was prepared as described before [8]. Briefly, Salmon fibrinogen and thrombin (Searun Holdings) were used for fabricating the 3D fibrin gel cell culture. The stock fibrinogen was diluted to 2 mg/mL with T7 buffer (50 mM Tris, 150 mM NaCl, pH 7.4). The fibrinogen and cell solution were mixed at a ratio of 1:1 to a final fibrinogen concentration of 1 mg/mL (required for 90 Pa gel). One thousand cells in the fibrinogen/cell solution mixture were seeded in each of the wells of thrombin-activated 96 well plates. The 96 well plate was placed into a 37 °C cell culture incubator for 15 min for gel polymerization. Finally, 200 µL of cell culture medium was added to every well. Cells were fed with fresh culture medium every two to three days. TRCs were isolated from 3D fibrin gels after ten days for experiments.

The sample holder for the Raman assay was fabricated by creating 5 mm diameter × 1.3 mm deep chambers in an aluminum plate. A Metrohm P-SERS (Printed-Surface Enhanced Raman Spectroscopy) silver substrate (Metrohm; 607506100) was cut into small pieces and placed inside the chamber of the aluminum plate. The chamber was incubated with 5 µg/mL fibronectin solution (Sigma-Aldrich; F1141-1MG) at 37 °C for at least 1 h for an efficient adsorption of fibronectin on the surface of the substrate. The substrate was subsequently washed with phosphate buffer saline (PBS) (Thermo Fisher; 1,0010-023). The parental control cells and TRCs were plated on the surface at a density of 5000 cells/substrate in a 30 µL droplet. The surrounding surface was covered with double-sided tape and a No. 0 glass coverslip was placed on top of the chamber to create a seal. Finally, the chamber was completely sealed off around the edge with Scotch^®^ tape to contain biohazard cellular materials. The samples were sealed to prevent the contamination of the samples, exposure of the user to the samples, and evaporation to the open air.

Despite the cells being anchored (fixed) on a substrate using a cell adhesion molecule (fibronectin), the spectral signature of fibronectin should very minimally affect the ability to discern the difference between TRCs and control cells, since the experimental designs and procedures of both TRCs and control cells were identical.

### 3.2. Data Collection

After the cells were harvested, they were analyzed using a modular Raman microscope system (Horiba iHR550 imaging spectrometer in conjunction with an Olympus BX41 microscope) immediately to minimize excessive cell decay. Near-infrared (NIR) light at a wavelength of ~785 nm (Toptica Photonics single-frequency laser iBeam-Smart-785-S-WS) was used as the excitation light source to minimize the autofluorescence from biological samples.

Many optimization procedures were addressed to improve the signal-to-noise ratio (autofluorescence) and reproducibility of the Raman signal. First, a SERS substrate that provided the maximum Raman signal was selected. Note that prior to the current experimental approach, a preliminary data collection was attempted using a spontaneous Raman technique (without a SERS substrate) and with gold and silver SERS substrates. Both of the SERS substrates allowed the procedure to perform several orders of magnitude stronger than without, where the enhancement factors can be as much as million-fold [26,27], and the silver substrate appeared to yield a better signal to noise ratio than the gold (more than an order of magnitude) [28]. Additionally, dominant Raman peaks (728 cm^−1^ (C-C stretching, proline (collagen assignment)), 1092 cm^−1^ (PO_2_^−D^), etc.) [29] used for optimization were determined by comparing the sample spectra to spectra of the SERS substrate alone and of the substrate with fibronectin but without cells, allowing these background contributions to be minimized in the sample spectra. Second, the distance between the sample and the objective lens was set to yield a maximum peak intensity. Finally, a suitable objective lens and depth of camber were set to minimize Raman emission from the glass coverslip.

The laser is relatively more stable in power and wavelength at a higher power configuration (~80 mW); however, the high intensity of laser light could potentially damage the samples after a prolonged and focused use in one spot, so a filter (~50%) was used at the microscope entrance to reduce the power to ~40 mW, and, as shown in Section 4, the results indicate that this power setting was not an issue.

As the cells are more likely to be on the fibronectin, the Raman probing locations on the sample were determined by locating the fibronectin using the charge-coupled device (CCD) camera of the Raman microscope. Owing in small part to the cells themselves being difficult to resolve with the current setup and without cell staining, preliminary data had been collected for the individual noncellular components used in the sample preparation as mentioned above. Optimization included looking for signals that were not attributable to the noncellular components of the sample.

The spectra were collected after signal optimization using a 20× magnification objective lens for ten 15 s scans over the desired range to produce one spectrum. Then the stage was moved laterally by the two horizontal axes, moving to other locations within the deposition area. This process was repeated for a total of at least three different spots on each of the 13 different samples before moving to the next.

### 3.3. Data Preprocessing and Analysis

**Figure 1:**
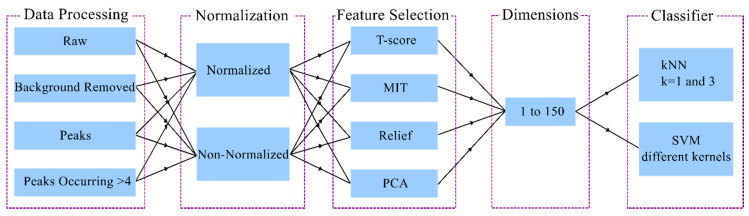
All possible variable combinations were used for the classification.

Different data types, feature reduction techniques, and classifier types were varied, where all possible combinations of these were investigated for normalized and non-normalized spectra (Figure 1).

To minimize the influence of any residual autofluorescence and enhance the signal to noise ratio, a baseline correction was implemented by fitting the baseline section-by-section with a semiautomated polynomial function using an in-house built program.

For raw and background-removed spectra, each wavenumber (dimension) had a measured value, but for the peak data, only the wavenumbers that corresponded to a local maximum had a nonzero value. If any spectrum had a peak at a particular dimension, then that wavenumber was used in the analysis. If the other spectra had a peak at that wavenumber the value was recorded; otherwise, a value of zero was assigned to that dimension.

## 4. Results and Discussion

**Figure 2:**
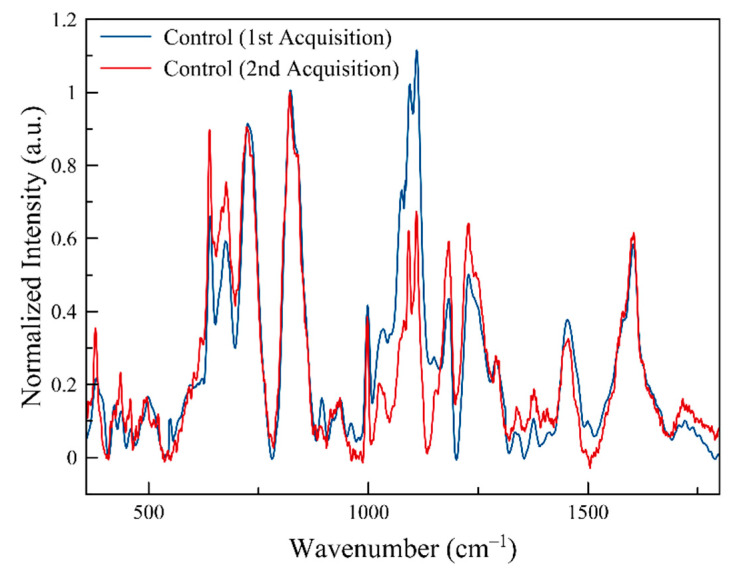
Two individual spectra acquired at the same location from a sample of control cells.

Prior to conducting this experiment, the nondestructive nature of Raman spectroscopy with small sample volumes exposed to focused laser light was first confirmed. Two successive spectra were acquired from control cells that were exposed to the laser light at the same location and under the aforementioned parameters (except dramatically increasing the actual exposure time to at least ~10 min) and no discernible changes were detected in the successive spectra beyond that attributable to the fluctuations or movements of cellular components within the cells and the inherent uncertainty of Raman scattering, as can be seen in Figure 2.

**Figure 3:**
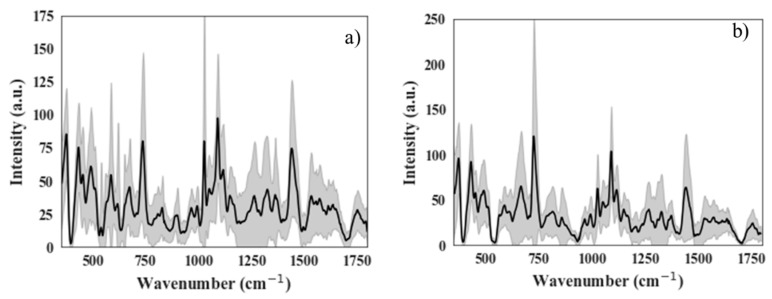
Average Raman spectra with shaded error bands: (**a**) Control Cells (**b**) tumor repopulating cells (TRCs).

**Figure 4:**
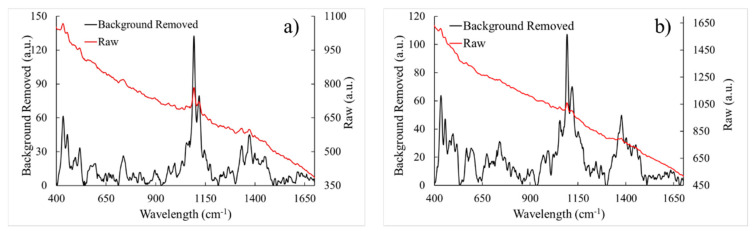
Raw and Background-Removed spectra of surface enhanced Raman spectroscopy (SERS) from a single location of a sample: (**a**) Control cells and (**b**) TRCs. Left axis for background removed and right axis for raw data.

**Figure 5:**
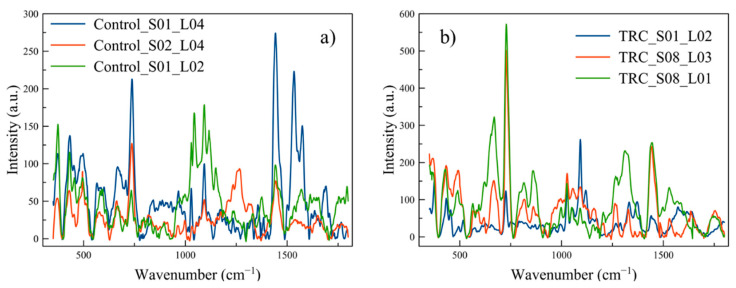
Comparison of three spectra from the same type of cells to demonstrate variation present within the same sample (S##) but with different location (L##) and for different samples (S##): (**a**) Control and (**b**) TRC.

For the initial results, 51 Raman spectra were collected from 13 samples: eight parental controls for 37 spectra and five TRCs for 14 spectra. The limited number of TRC samples was due to the long growth times (10 days) involved in producing these cells. Figure 3 shows the average Raman spectra of control cells and TRCs with shaded error bands (shown for clarity and to display the high variation in the Raman spectrum with measurements—averaged data was not used in the ML analysis), and Figure 4 shows a raw Raman spectrum and the background removed version of a single location for control cells and TRCs. Figure 5 shows three non-normalized instances of control and TRC Raman spectra to demonstrate the significant variation in spectra from the same class of cells.

**Figure 6:**
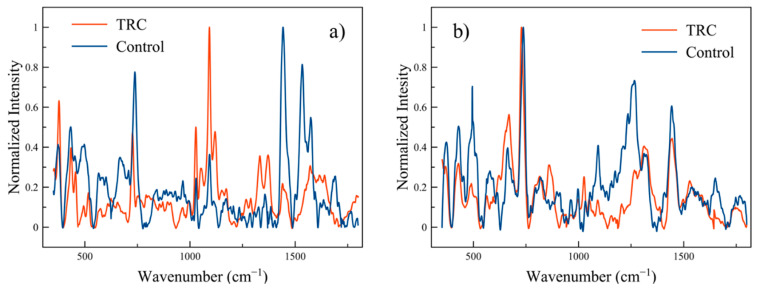
TRC and Control spectra normalization: (**a**) TRC and Control normalized to different peaks and (**b**) normalization to the same peak.

In Figure 6, normalized TRC and control cell spectra are compared. Figure 6a shows a case where the normalization is with respect to different peaks (the maximum peak values occurred at two different wavenumbers for TRCs and Controls), while Figure 6b shows the normalization over the same peak. This serves to demonstrate that there is no single peak with which to normalize consistently; therefore, all normalization in this work is to the maximum peak of each spectrum.

**Figure 7:**
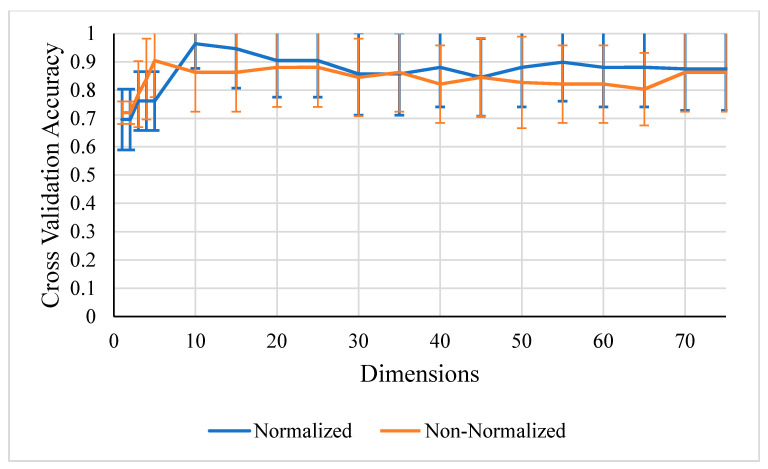
k-Nearest Neighbor (kNN) k = 1 with *w* selected peaks cross validation scores for 1 to 75 dimensions that demonstrate normalized data obtain better results with this classifier.

**Figure 8:**
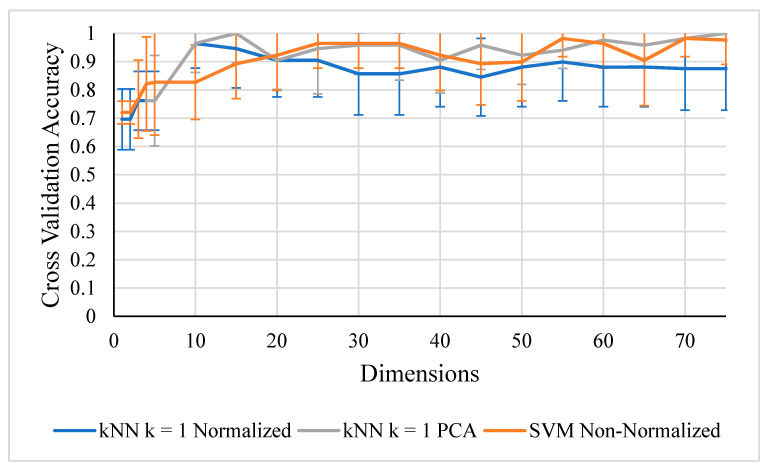
Shows normalized k-Nearest Neighbor (kNN) k = 1 underperforming non-normalized kNN + principal component analysis (PCA) and support vector machine (SVM) in the range of over 20 dimensions. All peaks selected with *w*.

The top classifier accuracies for 60 or fewer dimensions of non-normalized data are presented in Table 2. While Figure 7 demonstrates that normalization improved the kNN classification results, Figure 8 shows that non-normalized SVMs and kNNs with PCA both perform better in the range of 20 to 75 dimensions with less standard deviation. Since PCA includes normalization, any prior normalization would be redundant computationally. Normalization without PCA also caused the SVM algorithm to perform poorly, so normalization is not present in any of the best combinations of Table 2.

While all the top results in Table 2 are for peak data, the best background-removed data result had an accuracy of 0.85 with a sigmoid SVM for *w* + PCA. RELIEF did not perform as well as the other selection techniques with the best results for *r* + PCA with a sigmoid SVM on peak data yielding an accuracy of up to 0.93. An in-depth investigation that involves computational theories of data handling (binning), statistical analysis, and a study of matrix effects for peak shifts is required to understand why the peaks data performed better than the others in this work. This approach may lead to a better understanding and improve the performance of the data-driven spectroscopy in various fields, including for biomedical applications.

Table 2 also contains the training accuracy as an indicator of the overfitting of the models, as mentioned before. The shaded rows are runs where the training score exceeded the testing score, and this occurred for both *t* + PCA and *w* + PCA for the dimensions presented, but only for specific dimension counts.

In the results shown in Table 2, all the combinations, except one, achieved an accuracy of 0.982. This uniformity of results is not a sign of an error in the analysis, but is, instead, the result of the small sample size and the CV technique used. To allow for more involved metrics later on, the code was written to have at least one of each class in every test subset, so the 14 TRCs restricted the number of subsets for the CV (the option of leave one out cross-validation (LOOCV) was intentionally avoided in this work). With this CV configuration, each subset has two to three parental control cells and exactly one TRC. 

The 0.982 accuracy is from a 14-fold CV with 13 accuracies of 1.0 and one accuracy of 0.75 for a test subset of three parental control and one TRC. There were 34 combinations of tools that led to this accuracy. Five combinations had CV scores of 0.976 where there were 13 accuracies of 1.0, and a 0.6667 accuracy for a test subset with two parental control and one TRC. So, the small sample size and CV metric give the appearance of artificially static accuracies, but with a larger dataset, the apparent discreteness would be diminished.

With the feature selection tools, it would be interesting to look at the different combinations applied to the reduced dimensions already chosen. In other words, to find the best classifier results for a few hundred dimensions, and then run all the combinations on that reduced dataset. This would be especially interesting with *r*, as this method has been shown to do well at identifying artificial markers in other studies [18], but did not perform well here.

**Figure 9:**
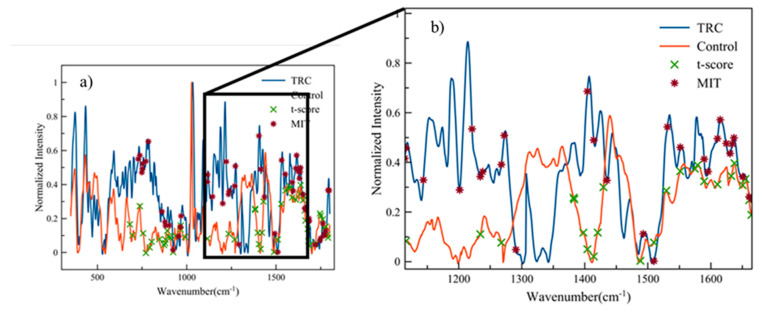
Raman spectra with t-score (*t*) and MIT (*w*) chose 55 dimensions. (**a**) Whole spectrum from 350 to 1800 m^−1^ and (**b**) zoomed-in region from 1115 to 1665 cm^−1^*. w* and *t* peaks are plotted over different curves for clarity.

After completing the data reduction and classification algorithms, the reduced dimensions for promising classifier results can be referenced to indicate which peaks led to the best results. Figure 9a shows the parental control and TRC spectra from 350 to 1800 cm^−1^ with markers for the dimensions chosen by *w* and *t* for the 55 dimension runs, as this is the region in Table 2 where the better reduction tool shifts from *t* to *w*. Figure 9b is a magnified section of the original, showing the region from 1115 to 1665 cm^−1^, while Table 3 represents a literature search for the possible causes of the selected peaks in Figure 9b.

The points in Figure 9b show signs of clustering of the Amide I and Amide III bands and also tyrosine and collagen selected as essential markers in distinguishing between Control and TRC cells, and this is illustrated in Table 3.

## 5. Conclusions

This work illuminates the potential of Raman spectroscopy in conjunction with statistical analysis and machine learning classification techniques to aid in the study of pancreatic cancer. The classification algorithms studied, with the chosen dimension reduction regimes, led to significant accuracy results in the comparison of parental control cells and TRCs.

It was shown that kNN and SVM techniques could both obtain an accuracy greater than 0.98 when differentiating the cell types with 35 to 60 dimensions of background removed and non-normalized, peak-selected data without overfitting of the models. SVM would seem to be a better candidate for further study, as it was able to achieve its accuracies without the addition of PCA, unlike kNN.

The practice of preserving the dimensions selected led to the observation of clustering in the biologically active Raman regions, which will lead to further targeting of the spectra, specifically in the 1100 to 1700 cm^−1^ region. This will increase the data collected in exciting regions while still reducing the overall dimensionality of the data and guide investigators toward the right questions to ask when looking at the biology within cells with different methods.

Important next steps will be to obtain more samples and spectra to track the behavior of the models as more TRC samples are made available, thereby allowing for a more balanced CV scoring of the classifier’s accuracy.

## Figures and Tables

**Table 1:** Statistical feature selection methods. The ${µ}_{j}^{k}$ are the mean of the ith feature for the kth class, and $\sigma_{j}^{k}$ are for the standard deviation of the same. In the RELIEF (*r*) algorithm the $x_{i}$ are the individual features.

Method	Description
t-statistic [14]	$t\left(x_{j}\right)=\frac{\left|{µ}_{j}^{0}-{µ}_{j}^{1}\right|}{\sqrt{\frac{{\left(\sigma_{j}^{1}\right)}^{2}}{n_{1}}+\frac{{\left(\sigma_{j}^{0}\right)}^{2}}{n_{0}}}}$
MIT Correlation [14]	$w\left(x_{j}\right)=\frac{\left|{µ}_{j}^{0}-{µ}_{j}^{1}\right|}{\sigma_{j}^{1}+\sigma_{j}^{0}}$
RELIEF [18]	$weight\left(x_{i}\right)=\left|x_{i}-miss\left(x_{i}\right)\right|-\left|x_{i}-hit\left(x_{i}\right)\right|$ $hit\left(x_{i}\right)=$ nearest neighbor of $x_{i}$ from same class $miss\left(x_{i}\right)=$ nearest neighbor of $x_{i}$ from opposite class
PCA	$z_{k}={\bar{\propto }}_{k}\cdot \bar{x}$ -reshapes the space into fewer dimensions capturing the maximum variance. ${\bar{\propto }}_{k}$ is the kth eigenvector of the covariance matric of $\bar{x}$, and $z_{k}$ is the projection of $\bar{x}$ in ${\bar{\propto }}_{k}$ dimension.

**Table 2:** Best Classifier Feature Selection using peaks for Low Dimensions. Highlighted rows show over-fitting.

Classifier	Reduction Method	Dimensions	CV Acc.	CV StdDev	Training CV	Training Stdev
SVM	t-score	35	0.982	0.064	0.911	0.018
kNN k = 1	t-score + PCA = 3	35	0.982	0.064	1	0
kNN k = 3	t-score + PCA = 3	40	0.982	0.064	0.980	0.005
kNN k = 1	t-score + PCA = 3	45	1	0	1	0
SVM	t-score	45	0.982	0.064	0.937	0.024
kNN k = 1	MIT + PCA = 3	45	0.982	0.064	1	0
kNN k = 1	t-score + PCA = 3	50	0.982	0.064	1	0
kNN k = 1	MIT + PCA = 3	55	0.982	0.064	1	0
SVM	MIT	55	0.982	0.064	0.876	0.024
kNN k = 3	MIT + PCA = 3	60	0.982	0.064	0.979	0.008

**Table 3:** Possible Molecular Source of Peaks from 1115 to 1665 cm^−1^ that were obtained from various literature references [29]. Note the grouping of Amide III and Amide I on either end of this range.

Wavenumber (cm^−1^)	T-Score	MIT	Possible Source	Reference
1116.4	X	X	CH_2,6_ in-plane bend and C_1_-C_α_-H_α_ bend	[30]
1201.5		X	Amide III (proteins) Amide III: C-N stretching and N-H bending	[31] [32,33]
1221.1		X	Amide III (β-sheet) Amide III (proteins)	[34] [31,35]
1234.2	X	X	A concerted ring mode	[36]
1237.9		X	Amide III & CH_2_ wagging: glycine backbone and proline side chains	[37]
1267.6	X	X	C-H (lipid in healthy tissue) Amide III (collagen assignment)	[33]
1272.3		X	CH$\alpha^{\prime }$ rocking	[30]
1290.7		X	Cytosine	[38]
1420.5	X		CH_2_ (lipid and protein) DNA/RNA Deoxyribose (B, Z-marker)	[35,39] [31] [38]
1488.2	X		Guanine (N_7_) Collagen	[38] [40]
1578.9	X		Guanine (N_3_) Guanine, adenine	[38] [31]
1610.4	X	X	Cytosine (NH_2_)	[38]
1614.8		X	Tyrosine	[41]
1634.0		X	Amide I	[37]
1637.5	X		Amide I	[42,43]
1650.5	X	X	Amide I	[33,44]
1654.9	X		Amide I C==C stretching Collagen	[34,37,45,46] [46] [47]
1660.9	X	X	Amide I C==C (lipids, fatty acids) Ceramide backbone	[31,48,49] [31,50,51] [51]
1664.4	X	X	Amide I	[41]
